# Nonviral Gene Therapy for Cancer: A Review

**DOI:** 10.3390/diseases6030057

**Published:** 2018-07-03

**Authors:** Chiaki Hidai, Hisataka Kitano

**Affiliations:** 1 Division of Physiology, Nihon University School of Medicine, 30-1 Oyaguchikami-cho, Itabashi-ku, Tokyo 173-8610, Japan; hidai.chiaki@nihon-u.ac.jp; 2 Division of Oral Surgery, Nihon University School of Medicine, 30-1 Oyaguchikami-cho, Itabashi-ku, Tokyo 173-8610, Japan

**Keywords:** cancer, gene therapy, nonviral vector, in vivo study

## Abstract

Although the development of effective viral vectors put gene therapy on the road to commercialization, nonviral vectors show promise for practical use because of their relative safety and lower cost. A significant barrier to the use of nonviral vectors, however, is that they have not yet proven effective. This apparent lack of interest can be attributed to the problem of the low gene transfer efficiency associated with nonviral vectors. The efficiency of gene transfer via nonviral vectors has been reported to be 1/10th to 1/1000th that of viral vectors. Despite the fact that new gene transfer methods and nonviral vectors have been developed, no significant improvements in gene transfer efficiency have been achieved. Nevertheless, some notable progress has been made. In this review, we discuss studies that report good results using nonviral vectors in vivo in animal models, with a particular focus on studies aimed at in vivo gene therapy to treat cancer, as this disease has attracted the interest of researchers developing nonviral vectors. We describe the conditions in which nonviral vectors work more efficiently for gene therapy and discuss how the goals might differ for nonviral versus viral vector development and use.

## 1. Introduction

Currently, cancer therapy around the world consists mainly of surgery, chemotherapy, radiotherapy, and multimodality therapy. However, as the progression of cancer still cannot be controlled, gene therapy is attracting increasing attention.

Gene therapy using viral vectors has recently been proven to be an efficient approach, as exemplified by excellent results obtained using viral-mediated cytotherapy to treat diseases caused by genetic deficiencies, a clear area of interest for gene therapy-based approaches [1,2]. Among such successes are lentiviral hematopoietic stem cell cytotherapy, used to treat metachromatic leukodystrophy, and viral vector-based approaches, used to treat Wiskott–Aldrich syndrome. In these approaches, the transduced gene is expressed at levels sufficient to give therapeutic value, and this expression can continue for several months or longer. One caveat to these studies is that because the number of cases was small and the observation periods were short, the possibility of side effects has not yet been fully evaluated. However, gene therapy has become a realistic choice for the treatment of diseases caused by genetic deficits [3].

Gene therapy is not limited to correcting genetic defects. A variety of gene therapy approaches have been attempted for the treatment of many kinds of diseases. Regarding nonviral vectors, safety is often mentioned as an advantage and inefficiency as a disadvantage [4,5]. Conversely, for viral vectors, efficiency is often discussed as an advantage, and immunogenicity, pathogenicity, and carcinogenicity as disadvantages. Over time, the emphasis on ensuring the safety of medical treatment has increased. From the point of view of safety, unless no other comparative alternatives, such as those used to treat genetic defect diseases, are found, there is no conclusive evidence to suggest that viral vectors will be used actively and broadly. It is impossible to deny the potential carcinogenicity, immunogenicity, and pathogenicity of viral-based and related approaches, including phage- or transposon-based approaches, and these issues have attracted increasing attention in recent years [6,7,8].

Owing to the unproven safety of viral vectors, nonviral vectors are not used, and the majority of researchers do not seem to be interested in them. We searched the literature and found that in 2017, papers related to nonviral vectors comprised only 0.24% of all articles on gene therapy. Moreover, although the number of publications per year on gene therapy has increased 1.5 times over the last 5 years, no change has been observed in the number of publications per year on nonviral vectors. The unpopularity of nonviral vectors is likely attributable to reports of their low transfer efficiency compared with viral vectors. Indeed, the gene transfer efficiencies of existing nonviral vectors appear to be too low for practical use. It seems possible that nonviral approaches might never be practical as gene therapy specifically targeting genetic deficiencies, but this does not mean that they are without potential value. This raises the question, what types of improvements might make nonviral approaches useful for some types of gene therapy?

In this review, we outline the current state of cancer gene therapy with nonviral vectors. We then introduce case studies where in vivo treatment using nonviral vectors resulted in a survival benefit in animal models of cancer, for which a high gene transfer efficiency is required. Analyzing successful examples of gene therapy with nonviral vectors might be helpful in identifying appropriate ways to use them therapeutically. We also refer readers to other reviews for information on new technologies and novel nonviral vectors currently in development [5,9,10,11].

## 2. Gene Transfer Efficiency of Nonviral Vectors

Several nonviral transfection methods, namely microinjection, electroporation, hydrodynamic injection, and magnetic microparticles, and even chemical methods (cationic lipoplexes), have been reported [12]. The most sterling and recommended nonviral gene transfer technology is nucleofection, which can directly transfer DNA to the nucleic acid in living cells with high transfection efficiency [13]. Investigations into the delivery of interfering RNA have helped to optimize stem cell management, and the trends observed in mediated nonviral gene delivery systems incorporating advanced strategies are promising [14].

Several studies have reported that nucleofection mediates gene transfer more efficiently than other nonviral methods with various resistances to cell transfection [15,16,17,18].

In addition, several RNA delivery systems continue to be used for RNA interference, including small interfering RNAs (siRNAs) and microRNA. The RNA delivery system mechanism was derived from classic gene transfer techniques. The objectives of RNA delivery approximate for plasmid transfection. When the messenger RNAs are delivered to the cell and translated, they offer the potential of protein expression [19,20]. Circular RNAs are endogenous with stable structures and highly tissue-specific expression [21].

Meanwhile, several studies have advocated the importance of plasmid size for transfection efficiency. Presumably, the nonviral vector design is of prime importance [22,23,24]. Increased electrostatic interaction is observed between nonviral vectors and negatively charged cytoplasmic membranes, which accelerates receptor-mediated endocytosis. Therefore, improving cellular uptake is the most common approach to enhance the efficiency of siRNA delivery [25].

The difference in gene transfer efficiency between viral and nonviral vectors is striking [26,27]. The efficiency of nonviral vectors is reportedly 1/10th to 1/1000th that reported for viral vectors. An example comes from a study by Hama et al. [28], who compared gene expression in vitro after treatment with adenovirus vector or Lipofectamine Plus, a representative nonviral vector. They found that Lipofectamine Plus was 1000 times less effective than the viral vector. Similarly, Varga et al. [29] compared the adenovirus vector and polyethylenimine (PEI), another representative nonviral vector, and found that PEI was 10 to 1000 times less effective than the adenovirus vector. These findings suggest that the use of nonviral vectors for gene therapy is unrealistic. Over the last several years, some progress has been made in addressing the low efficiency of nonviral vectors. Prior to the studies by Hama et al. [28] and Varga et al. [29], a great deal of effort was devoted to improving the cellular uptake of DNA. To increase cellular uptake, ligands such as folate, epidermal growth factor, or arginine-glycine-aspartic acid peptide have been used as components of vectors [30,31,32]. In the two studies mentioned above, the researchers determined the steps for which gene expression with nonviral vectors were significantly inferior compared with viral vectors. They found that DNA uptake into cells was better with nonviral than with viral vectors. Conversely, in subsequent steps, i.e., DNA release into the cytoplasm and entry into the nucleus, adenovirus was superior to nonviral vectors. With this in mind, further development of nonviral vectors came to be focused on improving the efficiency of the steps between DNA uptake and transcription.

A comparison of the efficiencies of nonviral and viral vectors provides useful guidance for the further refinement of nonviral approaches [33,34]. Gaining a better understanding of how viruses achieve high transduction efficiency should help improve nonviral vectors. To enable high gene transfer efficiency, instead of using the virus itself, it might be possible to generate reagents with the properties of viruses. For example, with some viral vectors, as DNA is transferred from the endosome to the cytoplasm, the low pH in endosomes appears to promote the fusion of the lipid membrane and virus. Inspired by this phenomenon, Sato et al. [35] developed a multifunctional envelope-type nano device cationized at a low pH that had 100 times greater effectiveness than conventional nano devices. The attention on the development of next-generation nanomedicines highlighted their dependency on the size of lipid nanoparticles for efficient penetration into tumor tissues [36,37].

A related approach for improving gene transfer efficiency is to harness the same endogenous cellular functions utilized by viruses. For example, most viruses, including adenoviruses, enter cells via endocytosis. It has been discovered that a fragment of Del1, an extracellular matrix protein, enhances endocytosis and gene transfer [38,39]. This function has been utilized to perform nonviral gene therapy [40]. The introduction of fragments or full-length proteins that are endogenous to the organism is presumably a safe approach that could be used repeatedly, as the proteins should not be toxic or immunogenic [40].

## 3. Efficiency of Gene Therapy with Nonviral Vectors

For which diseases have “powerless” nonviral vectors been studied? To date, 2597 gene therapy clinical trials have been conducted worldwide [41]. Compared with 2007, about 1300 more gene therapy clinical trials were conducted in 2017 [42]. Indeed, 559 of all gene therapy trials conducted in 2017 used nonviral approaches (http://www.wiley.co.uk/genmed/clinical). A search of PubMed for papers related to nonviral vectors revealed that the most common type of disease explored in the development of nonviral vectors was cancer, followed by cardiovascular disease [43,44,45]. It is therefore tempting to speculate that researchers chose common diseases. Below, we briefly discuss the use of nonviral vectors for the treatment of diseases other than cancer and then provide examples from the cancer treatment field for which nonviral vectors showed promise.

### 3.1. Gene Therapy for Diseases Other Than Cancer

As mentioned above, viral vector–based treatment of genetic diseases, such as that based on constitutive gene expression following genome integration, has met with some success. Although nonviral vectors have also been explored as a treatment for genetic diseases, the expression of introduced genes decreases rapidly, limiting the success of this approach [46]. Therefore, some approaches aiming to extend the window of expression have been developed. For example, the use of minicircle DNA, which lacks bacterial nucleotide sequences, can extend the duration of gene expression [47]. Inclusion of the hepatic locus control region, an intron, and an untranslated region increases and stabilizes hepatic factor IX gene expression in vivo [48]. However, the results remain inferior compared with those achieved using genome integration via viral vectors. This problem, the gene transfer efficiency of nonviral vectors, has been further addressed by the development of devices that allow for continuous injection of DNA deep into the body, such as into the liver [49,50]. Although these devices are clearly useful, typically, the treatment of genetic deficiencies must continue throughout life, such that nonviral delivery might never be as convenient or effective as viral-mediated integration of DNA into the genome. Recently, the applicability of gene and cell therapy has been tested. However, the use of hydrodynamic gene delivery approaches has been limited in clinics because of safety concerns and the lack of pancreas specificity [51].

Low transfer efficiency might be a problem for other types of diseases as well. The efficiency of gene transfer with transfection reagents is so low that in some cases, there is no discernable difference in the efficiency of gene transfer with transfection reagents versus the introduction of naked DNA. For example, Zabner et al. [52] reported that in experiments aimed at introducing DNA to treat cystic fibrosis using a transbronchial transmembrane conductance regulator, they could not observe any significant difference following the administration of cationic lipid lipoplexes versus naked plasmid DNA. However, despite a low transfection efficiency, nonviral vector-based gene therapies have shown some significant results in the treatment of diseases in model mice. For example, local injection of naked DNA encoding stromal cell derived factor-1 to myocardial infarction in rats resulted in improved cardiac function [53]. Moreover, Kwon et al. [54] reported that local injection of minicircle DNA encoding vascular endothelial growth factor, together with a cationic dendrimer, improved the wound healing process in a diabetic mouse model. In a study by Pizybyszewska et al. [55], cDNA encoding a soluble tumor necrosis factor receptor, together with PEI, injected intramuscularly into pulmonary fibrosis model mice once weekly for 10 weeks led to an improved prognosis. Recently, a high level of transfection and transduction efficiency was achieved by the Lipofectamine 3000 transfection reagent compared with Lipofectamine 2000 or FuGENE 6 reagents [56]. Taken together, these successful cases suggest that gene therapy with nonviral vectors might have potential, particularly if reagents for transfection, routes of administration, and target conditions are selected appropriately.

### 3.2. Gene Therapy for Cancer

Despite concerted efforts by many researchers, no studies showing more successful cancer gene therapy for the treatment of cancer patients using nonviral vectors have been reported [57,58]. However, nonviral gene therapy has the potential to impact the treatment of cancer patients. Further efforts are required to increase the clinical application of nonviral cancer gene therapy, however. The main recognized disadvantage of nonviral vectors, i.e., a low gene transfer rate, seems highly relevant in terms of eliminating cancer cells. Additionally, the functional characterization of nonviral delivery systems such as nanoparticles, liposomes, or dendrimers remains an important point [59]. The clinical success of cancer gene therapy with nonviral vectors appears to remain a distant goal.

To get a sense of the outlook for future development, we reviewed the literature from 2008 to the present and identified 17 studies using nonviral vectors in in vivo animal models of cancer. Among these reports, we were able to identify some studies reporting a significant improvement in survival. Learning what these approaches have in common might provide us with clues for effectively using nonviral vectors. Therefore, we discuss the reports in more detail below.

In our literature search, we found nine studies in which treatment of animal models with nonviral vectors led to an improved prognosis. Three of the nine studies were reported by Klutz et al. [60,61,62] and explored the treatment of neuroblastoma or hepatocellular carcinoma using a single approach. Specifically, in these studies, DNA encoding a sodium iodide symporter was administered by intravenous injection of nonviral vectors into mice with explanted tumors. Next, radioactive iodine was injected intravenously. Repeated treatment led to a significantly improved prognosis. Different types of nonviral vectors were used in each of these studies. It is interesting that no difference was found in terms of the effects observed following treatment with a pseudodendritic oligoethylenimine vector versus treatment with a vector composed of an epidermal growth factor receptor (EGFR) peptide. Even in the absence of target-directing components such as EGFR peptide, selective accumulation of DNA in a tumor can be obtained via enhanced permeability and retention of tumor blood vessels. The limitation of this approach is the radiation dose. However, the results are meaningful in that they demonstrate the potential of systemic administration of nonviral vectors for the treatment of cancer.

Sharma et al. [63] used a nanoparticle with lactic-co-glycolic acid to introduce the *p53* gene for the treatment of explanted prostatic cancer in a p53 null mouse. The tumor suppressor protein p53 is encoded by the tumor protein *p53* gene. Additionally, p53 plays a primary role in a number of signal pathways, including the cell cycle, proliferation, differentiation, and apoptosis [64,65]. They also tried systemic administration by intravenous injection, which led to a significant improvement in life prognosis; however, this was not as effective as a local injection. Target-directing components such as EGFR peptide were not included in their vector; nevertheless, selective accumulation of the DNA complex in the tumor was observed.

Sun et al. [66] examined the potential preventive effects of gene therapy aimed at preventing lung metastases by evaluating the effects of *p27* gene therapy using a nonviral gene delivery strategy on pulmonary metastatic tumors. Specifically, they tested the effects of systemic intravenous injection into colon cancer cells using the *p27kip* gene. They performed multiple rounds of directed combination therapy involving cisplatin and nonviral gene therapy. As for the Sharma et al. study [63], although target-directing components such as EGFR peptide were not included in the vector, selective accumulation of DNA complex in the tumor was observed. Gene therapy proved to be effective when combined with cisplatin treatment [66].

In the following studies, DNA was injected locally. Finoccharo et al. [67] tried to treat spontaneous melanoma in dogs using a combination of chemotherapy, gene therapy, and cytotherapy. Injection into a tumor of the thymidine kinase (TK) gene using a lipoplex method tended to result in an improved prognosis [68]. Combination therapy involving ganciclovir, interleukin-2, granulocyte macrophage colony stimulating factor (GM-CSF) cell therapy, and gene therapy was found to be significantly effective. They also had encouraging results after treating spontaneous sarcoma in dogs via injection into the tumor of the TK gene with ganciclovir and interferon-α [69]. This treatment led to a significant improvement in prognosis [68].

Casey et al. reported treatment of a fibrosarcoma model mouse by gene transfer using naked DNA encoding B7-1 and GM-CSF [70]. Both treatment approaches resulted in a significant improvement in prognosis, suggesting that tumor growth is therapeutically inhibited [70].

Fewell et al. [71] created a peritoneal dissemination model of ovarian cancer. Using polyethyleneglycol (PEG) and PEI, the interleukin-12 gene was repeatedly administered intraperitoneally. This treatment improved prognosis and was associated with minimal toxicity and an additional improvement in survival. Combining this approach with Taxol further improved prognosis.

Kitano et al. [40] used a multifunctional fragment from Del1, an extracellular matrix protein that induces apoptosis, localizes to the extracellular matrix, and increases gene transfer efficiency via nonviral vectors [38,72,73]. In their study, cDNA was injected intratumorally into an explanted squamous cell carcinoma using jet-PEI once weekly. This treatment led to a significant improvement in prognosis. Looking at the commonalities among these approaches can be informative. One commonality is that for these studies, the nonviral gene therapy targets were mainly refractory cancers such as malignant melanoma, neuroblastoma, sarcoma, metastatic cancer, or peritoneal dissemination. The standard cancer treatment methods were surgical therapy, chemotherapy, and radiation therapy. Surgery and radiation therapy are generally effective for early stage cancer, such that in these cases, there is no demand for gene therapy. However, currently, cancer gene therapy should be limited to patients who are not candidates for surgery (e.g., because of metastasis), chemotherapy (e.g., because of drug resistance), or radiation therapy (e.g., because of limits of radiation).

It is essential to discuss the routes of administration of DNA used in these studies. As reported by Sharma et al. [63], local injection was more effective than systemic administration of a nonviral vector. Administration into a closed lesion, such as intraperitoneal or intratumoral injection, makes it possible to increase the concentration of the DNA complex, which seems to be helpful given the low gene transfer efficiency of nonviral vectors. Indications for gene therapy following local injection might exist. For example, liver cancer with intrahepatic metastasis is treated by either local infusion of ethanol or percutaneous radiofrequency ablation [74,75]. Local gene therapy might be an alternative to these types of treatments. In addition, it might be possible to apply gene therapy to the treatment of disseminated cancer present in the peritoneal or thoracic cavity. Moreover, endoscopic injection of DNA might be appropriate for patients with obstructive tumors in airways or the gastrointestinal tract who have already been treated with the maximum dose of radiation therapy.

We identified some reported cases for which the systemic administration of nonviral vectors was effective. It is interesting that DNA complexes accumulate in tumors without target-directed ligands, presumably because of the enhanced permeability and retention made possible by tumor vessels [76]. For intravenous administration, the incorporation of PEG into the vector is effective for increasing the retention of the DNA complex in blood [5]. PEG enhances permeability and retention; however, PEG also notably reduces uptake of the DNA complex by cells (the so-called “PEG dilemma”) [77,78]. To address this, a construct composed of a peptide-based connector that bridges a phospholipid and PEG and can be degraded in the tumor by a matrix metalloproteinase has been developed [79]. With this approach, the intact vector can enter a tumor via PEG, and then the processed vector (i.e., without PEG) can enter cells. The development of vectors with a complex function has led to an improvement in gene expression levels following systemic administration. On the other hand, considering the differences between systemic and local administration in pharmacokinetics, it may prove useful to develop different vectors for systemic versus local administration.

One of the simplest approaches to increasing the level of transgene expression is to perform repeated rounds of gene therapy. This is a realistic way to make use of the advantages of nonviral vectors, including safety in the cases of both local and systemic injection. Repeated intravenous administration of a small amount of a DNA complex could enhance accumulation in a tumor without leading to the side effects associated with gene transfer and transfection into healthy tissue.

A previously reported method leading to the accumulation of expressed proteins in the extracellular matrix also appears to be effective [40]. In such an approach, fusion between a therapeutic protein and a “deposition domain” from an extracellular matrix protein leads to the posttranslational deposition of the fusion protein into the extracellular matrix. This approach can lead to an increase in the local concentration of the protein and extend the effective period in those cases for which the expression level is low. It can also suppress the effects of the protein on nontarget tissues.

Ultimately, the success of cancer gene therapies largely depends on the choice of genes to be expressed. Nonviral vectors cannot realistically be used to introduce DNA into all cancer cells. Thus, treatments with autonomously acting “anti-tumor genes” such as p53 or TK might not be appropriate routes of development when it comes to nonviral vectors. Innovation in the field depends on new ideas regarding how to compensate for the weaknesses identified for nonviral vectors. In the studies described above, proteins originating from transfected cells appear to exert positive therapeutic effects on surrounding untransfected cells. Choosing proteins that can change the cancer microenvironment is common among successful studies using nonviral vectors.

Recently, cancer stroma has been reported to play important roles in proliferation and metastasis [80,81]. Factors secreted from cancer cells activate cells in the stroma, and then activated stromal cells stimulate cancer cells to proliferate and migrate [82,83]. Therefore, it might be possible to treat cancer by manipulating the cancer microenvironment [84,85]. Approaches aimed at manipulating stroma, such as through the introduction of IL2, IL12, or GM-CSF, appear to contribute to the success of gene therapy by nonviral vectors. The biological features of these cytokines are likely to contribute to the observed success. For example, cytokines can function at low concentrations (i.e., in the range of pmol/L). This compensates for the low transfection efficiency and small amount of protein expression associated with nonviral vectors. From an identical point of view, the use of a Del1 fragment can also serve as a type of microenvironment manipulation. The radiation emitted from radioactive iodine, which accumulates in cells expressing the sodium iodide symporter, may also affect the surrounding cells.

## 4. Conclusions

Commonalities among successful studies with nonviral vectors include local injection, repeated administration, and the use of proteins that either modify the cellular microenvironment or function well at low concentrations. For cancer and other diseases, experimental systems with these characteristics have worked well. Therefore, it appears that nonviral vector–mediated gene therapy is suitable for some therapeutic approaches.

Nonviral vectors should be used in protocols tailored to their specific strengths and weaknesses. These protocols are likely to be different from those considered appropriate for viral vectors. Viral vectors appear to be uniquely well suited for the treatment of genetic defects. It might be useful to think about nonviral vector-based treatments in the same manner that we think about drug treatments. With drug treatments, the types of drugs and approaches to their administration are chosen according to the nature and condition of the disease. We propose that the targets and protocols for gene therapy using nonviral vectors should be chosen accordingly. With this in mind, we suggest that in medicine, nonviral vectors should not be compared with viral vectors. Instead, nonviral vectors should be compared to drugs, such as in terms of their medical and economic efficiencies.

Gene therapy performed via the nonviral vector-mediated introduction of a cDNA encoding an endogenous protein is effective, safe, and economical. Physiologically active substances are more effective and safer than novel chemicals, and manufacturing DNA is relatively inexpensive. In addition, the stability of DNA is likely to reduce the costs associated with transportation and storage; for example, Sun et al. [66] reported that a lyophilized lipid polycation DNA complex could be used for gene therapy after rehydration. Moreover, in a sense, DNA is custom-made by nature to be safe, stable, efficient, and versatile. The injection of erythropoietin or GM-CSF is currently performed to treat anemia and granulocytopenia, respectively. Currently, expensive protein replacement is used; however, if safe and effective nonviral vectors can be developed, then nonviral gene therapy might prove to be a more economical approach [86,87]. In general, we feel that the development of nonviral vectors could enable gene therapy to be the method of choice for a variety of diseases treated in the clinical setting. Therefore, the availability of effective nonviral vectors could have a major impact on the development of new treatments.
